# Inherited genetic variants associated with glucocorticoid sensitivity in leukaemia cells

**DOI:** 10.1111/jcmm.15882

**Published:** 2020-10-01

**Authors:** Tamao Shinohara, Kevin Y. Urayama, Atsushi Watanabe, Koshi Akahane, Kumiko Goi, Meixian Huang, Keiko Kagami, Masako Abe, Kanji Sugita, Yukinori Okada, Hiroaki Goto, Masayoshi Minegishi, Shotaro Iwamoto, Takeshi Inukai

**Affiliations:** ^1^ Department of Pediatrics School of Medicine University of Yamanashi Chuo Japan; ^2^ Department of Social Medicine National Center for Child Health and Development Tokyo Japan; ^3^ Graduate School of Public Health St Luke’s International University Tokyo Japan; ^4^ Department of Statistical Genetics Osaka University Graduate School of Medicine Osaka Japan; ^5^ Hematology/Oncology and Regenerative Medicine Kanagawa Children’s Medical Center Yokohama Japan; ^6^ Tohoku Block Center Japanese Red Cross Society Sendai Japan; ^7^ Department of Pediatrics Mie University Graduate School of Medicine Tsu Japan

**Keywords:** acute lymphoblastic leukaemia, genome‐wide association studies, glucocorticoid sensitivity

## Abstract

Identification of genetic variants associated with glucocorticoids (GC) sensitivity of leukaemia cells may provide insight into potential drug targets and tailored therapy. In the present study, within 72 leukaemic cell lines derived from Japanese patients with B‐cell precursor acute lymphoblastic leukaemia (ALL), we conducted genome‐wide genotyping of single nucleotide polymorphisms (SNP) and attempted to identify genetic variants associated with GC sensitivity and *NR3C1* (GC receptor) gene expression. IC50 measures for prednisolone (Pred) and dexamethasone (Dex) were available using an alamarBlue cell viability assay. IC50 values of Pred showed the strongest association with rs904419 (*P* = 4.34 × 10^−8^), located between the *FRMD4B* and *MITF* genes. The median IC50 values of prednisolone for cell lines with rs904419 AA (n = 13), AG (n = 31) and GG (n = 28) genotypes were 0.089, 0.139 and 297 µmol/L, respectively. For dexamethasone sensitivity, suggestive association was observed for SNP rs2306888 (*P* = 1.43 × 10^−6^), a synonymous SNP of the *TGFBR3* gene. For *NR3C1* gene expression, suggestive association was observed for SNP rs11982167 (*P* = 6.44 × 10^−8^), located in the *PLEKHA8* gene. These genetic variants may affect GC sensitivity of ALL cells and may give rise to opportunities in personalized medicine for effective and safe chemotherapy in ALL patients.

## 1 INTRODUCTION

Pharmacogenomics focuses on understanding the role of genetic variation on intra‐individual differences in drug efficacies and toxicities.^1^, ^2^, ^3^ Genome‐wide association studies (GWAS) have consistently shown evidence that genetic variants are associated with drug metabolism^4^, ^5^ and toxic responses to chemotherapy^6^, ^7^, ^8^, ^9^, ^10^, ^11^, ^12^ in paediatric acute lymphoblastic leukaemia (ALL) patients. As the majority of germline genetic variants of normal cells are also represented in the leukaemia cells,^2^ it is plausible that inherited variants may influence the drug sensitivity phenotypes of leukaemia cells. In addition to genetic polymorphisms, somatic mutations are also known to alter the drug sensitivity of leukaemia cells.^2^

Glucocorticoids (GC) are important agents for ALL chemotherapy.^1^, ^2^ In the absence of the ligand, the GC receptor (GR) forms an inactive complex with heat shock proteins and several immunophilins in the cytosol.^2^ However, when GC binds to the GR by passive diffusion, the GC‐GR complex translocates to the nucleus. As a result, the GC‐GR complex acts as a transcriptional factor and regulates the expression of downstream target genes, which induce apoptotic cell death. Thus, the gene expression level of GR (*NR3C1*) plays an essential role in GC sensitivity of ALL cells. In addition to the functional GRα isoform of the human *NR3C1* gene, there are multiple isoforms and post‐translational modifications.^13^, ^14^

It is clear that there is significant inter‐individual variability in GC sensitivity among childhood ALL patients. Early treatment response to GC mono‐therapy is one prognostic factor.^15^, ^16^ Moreover, in vitro GC resistance of primary cultured leukaemic cells of patients is one risk factor for treatment failure.^17^, ^18^ Gene expression level of *NR3C1* splicing variants, and resulting GC sensitivity, could be affected by genetic variation that alters promoter activity, mRNA stability and splicing efficiency. In addition to *NR3C1* genetic variation, other genes involved in both the inactive cytosolic complex and the downstream pathway(s) of the active GC‐GR complex may also be associated with the between‐patient variability in GC sensitivity. Although GC sensitivity of ALL cells, both in vivo and in vitro, has been previously examined, the molecular basis to this heterogeneity in response remains poorly understood.

We previously examined the influence of splice variants of the *NR3C1* gene coding region on the sensitivity to GC among a large panel of B‐cell precursor ALL (BCP‐ALL) cell lines.^19^ Using 72 leukaemic cell lines established from Japanese BCP‐ALL patients, comprehensive real‐time reverse transcription polymerase chain reaction (RT‐PCR) analyses were performed to examine all five *NR3C1* splicing variants. We observed the strongest correlation between GC sensitivity and two real‐time RT‐PCR analyses, both specific for *GRα* and *GRγ* isoforms. In contrast, no significant correlation was observed between GC sensitivity and the *GRγ* isoform. Our previous observations demonstrated that *GRα* is highly involved in the anti‐leukaemic activity of GC in BCP‐ALL. We further examined possible associations between GC sensitivity and eight known candidate single nucleotide polymorphisms (SNPs)^20^, ^21^, ^22^, ^23^, ^24^, ^25^ that are located in the *NR3C1* gene in 72 BCP‐ALL cell lines.^19^ An association was observed for rs72555796 which maps to the 5′ flanking area of exon 1A and showed marginal association with *NR3C1* gene expression level. We further performed a luciferase assay to verify the significance of rs72555796 on promoter activity, but a clear link was not observed.^19^ These observations exemplify the limitations of the candidate SNP approach and highlight the potential utility of a more comprehensive assessment of genetic variation through a genome‐wide investigation.

Here, in 72 BCP‐ALL cell lines derived from Japanese patients, we conducted genome‐wide SNP genotyping in a unique attempt to identify genetic variants associated with two outcomes, GC sensitivity and gene expression level of *NR3C1*.

## 2 MATERIALS AND METHODS

### 2.1 Leukaemic cell lines and GC sensitivity measurements

Seventy‐two leukaemic cell lines were derived from BCP‐ALL samples in Japanese patients.^19^ Represented in these cell lines included Philadelphia chromosome‐positive (Ph+, n = 14), *MLL*‐rearranged (*MLL*+, n = 10), t(1;19)‐positive (n = 13), t(17;19)‐positive (n = 3), t(12;21)‐positive (n = 2) and those categorized as 'B‐other' which were negative for all of the above representative five translocations (n = 30). Each cell line was cultured in RPMI1640 medium supplemented with 10% foetal calf serum (FCS). KOPN, KOCL, YAMN and YACL series of cell lines were established (1980‐2011) in our laboratory.^19^ The YCUB and KCB series were established at Yokohama City University and Kanagawa Children's Medical Center^26^ (provided by H. Goto in 2014). THP series, L‐KUM and L‐ASK were established at Tohoku University^27^ (provided by M. Minegishi in 2014). The MB series were established at Mie University Graduate School of Medicine^28^ (provided by S. Iwamoto in 2014). SU‐Ph2^29^ was established at Kinki University School of Medicine (provided by Dr Y. Maeda in 2010). TCCY^30^ was established at Tochigi Cancer Center (provided by Dr Y. Sato in 2011). HALO1^31^ and SK9^32^ were established at Tokyo Medical University (provided by Dr T. Look in Dana‐Farber Cancer Institute in 1997 and Dr S. Okabe in 2012, respectively). Endokun^31^ was established at Iwate Medical University, Morioka (provided by Dr M. Endo in 1997). Kasumi2^33^ was established at Hiroshima University (provided by Dr T. Inaba in 2010). SCMCL1 and SCMCL2^34^ were established at Saitama Children's Medical Center (provided by Dr J. Takita in 2014). P30/OHK^35^ and Nalm27^36^ were purchased from ATCC in 2012.

For each cell line, the IC50s (defined as the concentration of substance required to kill 50% of the cells in vitro) were previously estimated for prednisolone (Pred) and dexamethasone (Dex) (Sigma‐Aldrich) using an alamarBlue cell viability assay (Bio‐Rad Laboratories). Detailed methods and data have been previously reported.^19^ Briefly, 1‐4 × 10^5^ cells were plated into a 96‐well flat‐bottom plate in triplicate and were incubated in the absence or presence of seven concentrations of Pred or Dex. After further incubation with alamarBlue, absorbance at 570 nm was measured by a microplate spectrophotometer. Viability of the treated cells was estimated by the ratio: optical density of treated wells over the optical density of untreated wells.

### 2.2 Real‐time RT‐PCR analyses

Total RNA was extracted using TRIzol reagent (Invitrogen). Reverse transcription was performed with Superscript II reverse transcriptase (Invitrogen) and a random hexamer (Amersham Bioscience). Each sample was treated with RNase (Invitrogen). Real‐time RT‐PCR analysis was performed with a SYBR Green PCR Master Mix (Applied Biosystems). Gene expression level of *NR3C1* was evaluated with primers specific for exon 8‐exon 9a of the *NR3C1* gene that can detect *GRα*, *GRγ* and *GR‐A* for which detailed methods and data have been previously reported.^19^ Briefly, the forward primer of the exon 8 was 5′‐GAGGGAAGGAAACTCCAGCC‐3′, whereas the reverse primer of the exon 9a was 5′‐TCAGCTAACATCTCGGGGAA‐3′.^19^ GC‐sensitive KOPN39 (IC50 of Pred; 0.014 µmol/L, IC50 of Dex; 1.2 nmol/L) was used as a control. For an internal control, the *ACTB* gene expression was quantified using the forward primer, 5′‐ACCTTCTACAATGAGCTGCGT‐3′, and the reverse primer, 5′‐GTACATGGCTGGGGTGTTGA‐3′.^19^

To quantify the gene expression levels of *FRMD4B*, *MITF* and *TGFBR3* (the identified targets of this study), each cell line was cultured in the absence or presence of 100 nmol/L of Dex for 12 hours. Real‐time RT‐PCR was performed with cDNA of KOPN84 as a control, which is GC‐sensitive cell line (IC50s of Pred and Dex were 0.032 µmol/L and 1.44 nmol/L, respectively). Forward primers of *FRMD4B*, *MITF* and *TGFBR3* were 5′‐CACTTTTCCTGGGCAGCGAT‐3′, 5′‐AATACGTTGCCTGTCTCGGG‐3′ and 5′‐TCCATGGTCTGGACACCCTA‐3′, respectively, whereas reverse primers were 5′‐CAGCATTTCTCTGGACTGCC‐3′, 5′‐GAAGGTTGGCTGGACAGGAG‐3′ and 5′‐GCTGTCTCCCCTGTGTGAG‐3′, respectively. *ACTB* was quantified as an internal control.

### 2.3 Genotyping and quality control

Genotyping of each cell line was performed after approval from the ethics committee of the University of Yamanashi. Each cell line was genotyped using the Illumina Infinium OmniExpress‐24 BeadChip (San Diego, CA; numbers of SNPs; approximately 700 000). For genotype data quality control, SNPs were excluded from further analyses if the minor allele frequency was <0.01, the distribution of genotypes showed clear deviation from Hardy‐Weinberg equilibrium (HWE; *P* < 1 × 10^−6^), or the genotype call rate was <99%. No cell line samples were excluded based on genotyping success rate of below 95% and relatedness in an identity‐by‐descent analysis (PI_HAT > 0.35). After quality control procedures, data for a total of 331 281 SNPs in 72 BCP‐ALL cell lines were available for analysis.

### 2.4 Statistical analysis

The associations between SNP and IC50 values for Pred and Dex sensitivities and *NR3C1* expression were examined using linear regression assuming a log‐additive genetic model. Drug sensitivity IC50 values were log‐transformed for distribution normalization. In addition, genome‐wide association analyses were performed with logistic regression after categorizing cell lines into Pred‐resistant (n = 38, IC50 value of Pred > 2.8 μmol/L) and Pred‐sensitive (n = 34) and into Dex‐resistant (n = 34, IC50 value of Dex > 200 nmol/L) and Dex‐sensitive (n = 38). These cut‐points were based on previous reports of *C*_max_ showing 0.6‐1.4 µmol/L for Pred during remission induction therapy^37^ and 20‐200 nmol/L for Dex during re‐induction therapy^38^ in childhood ALL patients. *P* value of less than 5 × 10^−8^ was considered statistically significant at the genome‐wide level. SNPs with a *P* value less than 10^−5^ were considered to show suggestive associations.^37^ Analyses were performed with PLINK version 1.07 (http://pnguweb.mgh.harvard.edu/‐purcell/plink/), R version 3.6.0 and SAS software version 9 (SAS). Manhattan plots were generated using R package “qqman version 0.1.4”.

### 2.5 Bioinformatic evaluation

Gene expression levels in clinical samples of paediatric BCP‐ALL patients retrieved at diagnosis (n = 566) and at relapse (n = 77) using RNA sequencing data were accessed from St. Jude Cloud PeCan (https://pecan.stjude.cloud/). Gene expression levels in samples by treatment outcome, no relapse (n = 29) or relapse (n = 167), were also obtained through the Therapeutically Applicable Research to Generate Effective Treatments (TARGET, https://ocg.cancer.gov/programs/target) initiative, phs000464. Differences in gene expression levels at the identified gene candidates were evaluated.

## 3 RESULTS

### 3.1 Association between genetic variants and prednisolone sensitivity

**Figure 1:**
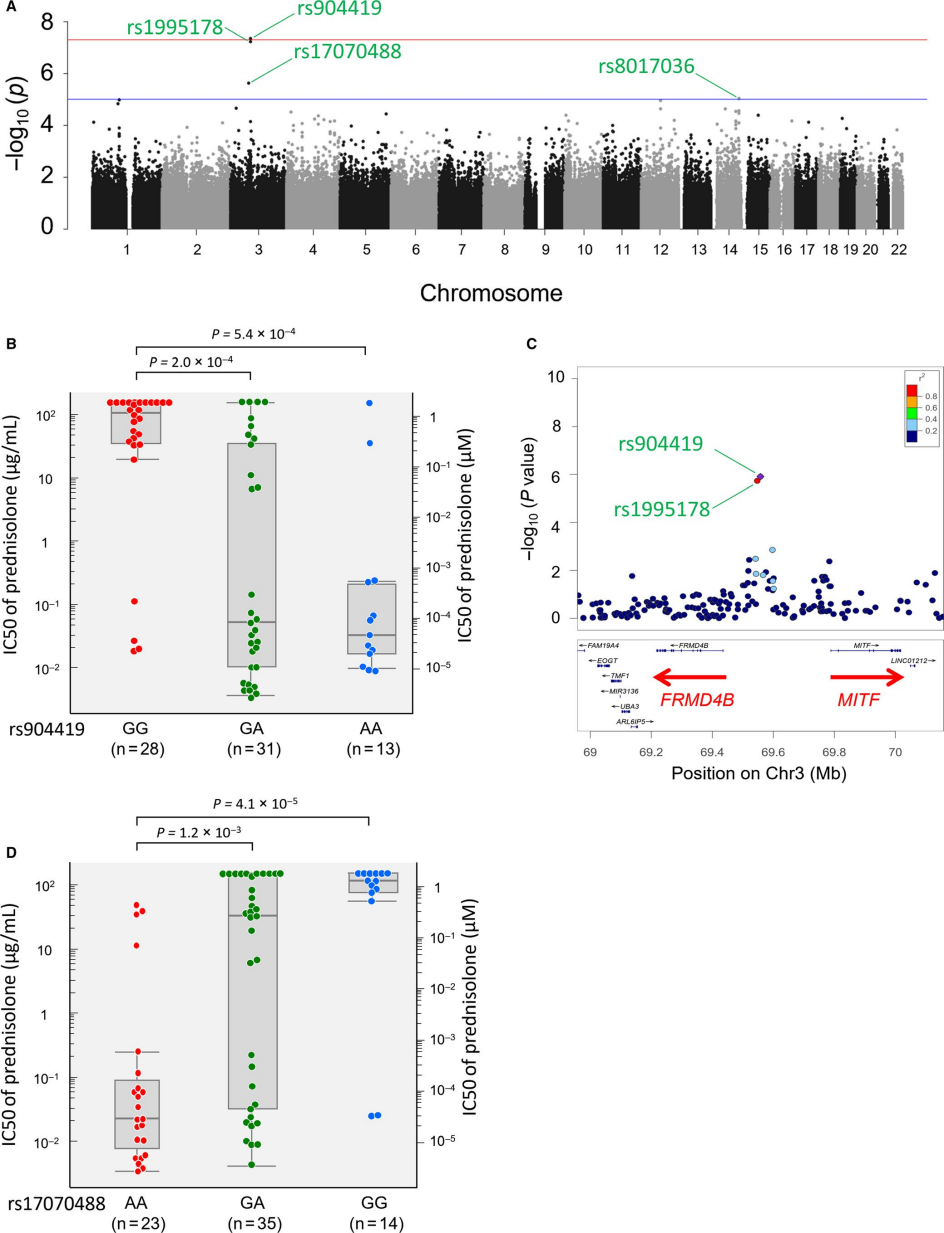
Association between inherited genetic variants and sensitivity to prednisolone

The range of IC50s for Pred sensitivity was 0.0092‐>417 μmol/L.^19^ Overall, genome‐wide association analysis for Pred log IC50 values showed minimal evidence of genomic inflation (λ = 1.065). SNPs representing four regions showed *P* values below the suggestive threshold (*P* = 10^−5^),^39^ of which one SNP in close 5′ upstream proximity to the *FRMD4B* gene located at 3p14.1 had genome‐wide significance (rs904419, *P* = 4.34 × 10^−8^; Figure 1A and Table 1). For rs904419, the median Pred IC50 values for cell lines with AA (n = 13), AG (n = 31) and GG (n = 28) genotypes were 0.089, 0.139 and 297 μmol/L, respectively (Figure 1B). The second SNP, rs1995178 (*P* = 5.76 × 10^−8^), was in strong linkage disequilibrium (LD) with rs904419 (*r* ^2^ = 0.971) and is located about 10 kb 3′ downstream of rs904419. rs904419 and rs1995178 are located between the *FRMD4B* and *MITF* genes (Figure 1C). A third SNP, rs17070488 located at 3p14.1 and approximately 5 megabases (Mb) away from the *FRMD4B* and *MITF* genes, showed a suggestive association (*P* = 2.29 × 10^−6^). Median Pred IC50 values for cell lines with GG (n = 14), GA (n = 35) and AA (n = 23) genotypes were 319, 90 and 0.061 μmol/L, respectively (Figure 1D). rs17070488 is located between the *PRICKLE2* gene and the long non‐coding RNA *ADAMTS8‐AS1*. A fourth SNP, rs8017036 located at 14q32.2 and annotated to the non‐coding RNA *LOC105370657*, also showed a suggestive association (*P* = 9.39 × 10^−6^).

**Table 1:** Top SNPs associated with prednisolone sensitivity

SNP	Chr	Position^a^	*P* value	Gene	SNP function	Minor allele (JPT)	MAF	
							JPT^b^	Cell line^c^
rs904419	3	69557771	4.34 × 10^−8^	*FRMD4B*	5upstream	A	0.395	0.396
rs1995178	3	69547173	5.76 × 10^−8^	*FRMD4B*	5upstream	T	0.395	0.389
rs17070488	3	64340527	2.29 × 10^−6^	*PRICKLE2*	intron variant	G	0.433	0.438
rs8017036	14	99016766	9.39 × 10^−6^	*LOC105370657*	downstream variant	A	0.349	0.382
rs12751763	1	96997655	1.05 × 10^−5^			T	0.395	0.417

^a^ Physical location of SNPs based on human genome build 37 (GRCh37).

^b^ Minor allele frequency in normal Japanese of the 1000 Genomes database.

^c^ Minor allele frequency in 72 BCP‐ALL cell lines established from Japanese patients.

The genome‐wide association analysis dichotomizing cell lines into Pred‐resistant (IC50 value of Pred > 1.4 μmol/L) and Pred‐sensitive cell lines (IC50 value of Pred < 1.4 μmol/L) resulted in no associated SNPs. However, rs904419 also showed to be the top SNP (*P* value; 3.1 × 10^−5^) in this analysis as well (Figure S1).

### 3.2 Association between genetic variants and dexamethasone sensitivity

**Figure 2:**
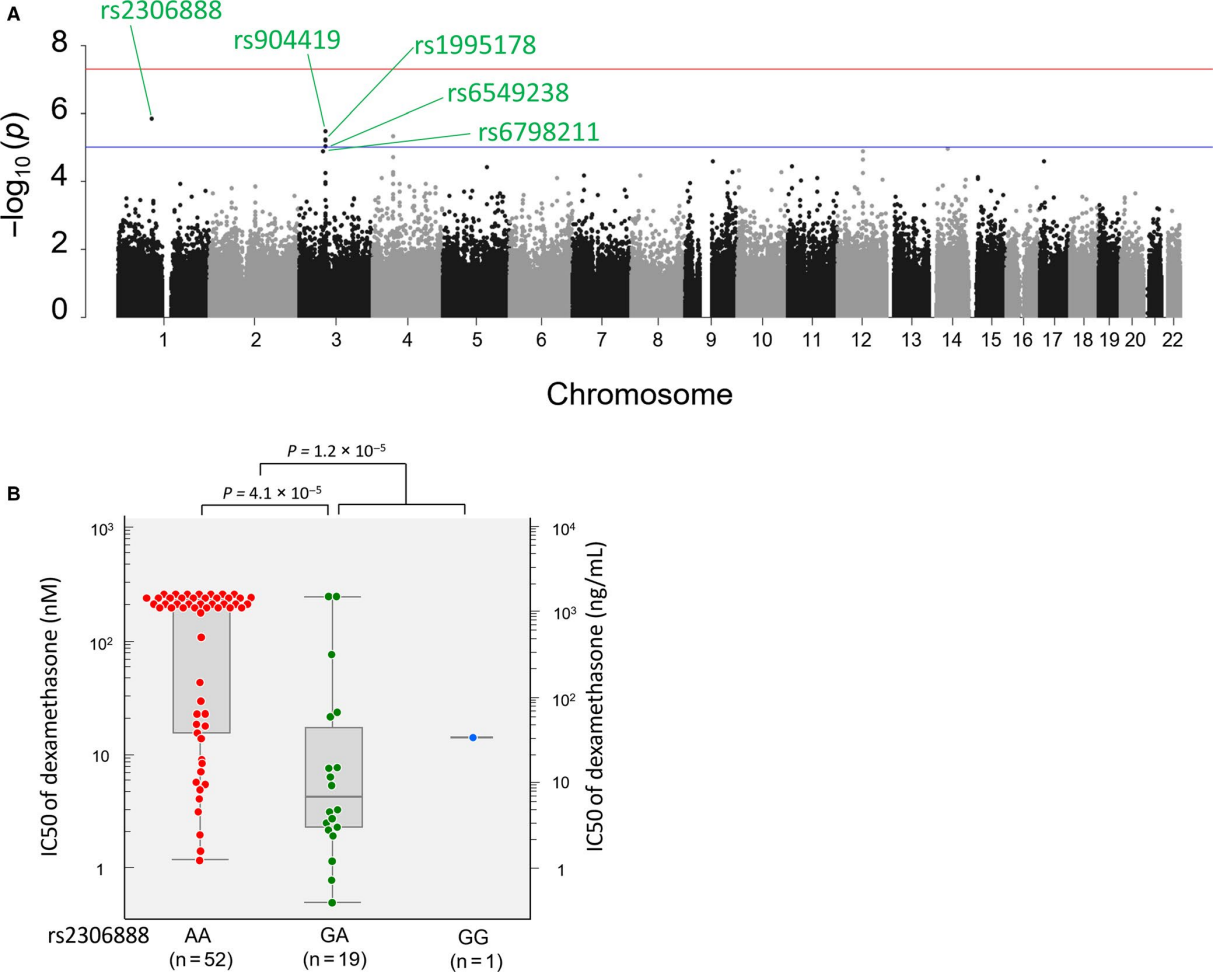
Association between inherited genetic variants and sensitivity to dexamethasone

The range of IC50s for Dex sensitivity was 0.48‐>250 nmol/L, and the values appeared correlated with those of Dex.^20^ Results of the genome‐wide association analysis showed minimal evidence of genomic inflation (λ = 1.061). Statistically significant associations were not observed, but six SNPs were suggestive at a *P* value of less than 1.0 × 10^−5^ (Figure 2A and Table 2). The strongest signal for potential association was observed on chromosome 1 for rs2306888 (*P* = 1.43 × 10^−6^) located at 1p22.1 and annotated to the *TGFBR3* gene (Figure 2A). This SNP also showed a low *P* value in the analysis of Pred sensitivity (*P* = 1.46 × 10^−5^). Median IC50 values of Dex for cell lines with rs2306888 GG (n = 1), GA (n = 19) and AA (n = 52) genotypes were 14, 3.2 and >250 nmol/L, respectively (Figure 2B). There were four suggestive SNPs (rs904419, rs1995178, rs6549238 and rs6798211) located at 3p14.1 which annotated to the *FRMD4B* gene, similar to the analysis of Pred IC50.

**Table 2:** Top SNPs associated with dexamethasone sensitivity

SNP	Chr	Position^a^	*P* value	Gene	SNP function	Minor allele (JPT)	MAF	
							JPT^b^	Cell line^c^
rs2306888	1	92200382	1.43 × 10^−6^	*TGFBR3*	nc transcript variant	C	0.186	0.146
rs904419	3	69557771	3.22 × 10^−6^	*FRMD4B*	5upstream	A	0.395	0.396
rs11133333	4	55380471	4.60 × 10^−6^			T	0.325	0.326
rs1995178	3	69547173	5.94 × 10^−6^	*FRMD4B*	5upstream	T	0.395	0.389
rs6549238	3	69597148	6.15 × 10^−6^	*FRMD5B*	5upstream	A	0.235	0.257
rs6798211	3	69588295	9.19 × 10^−6^	*FRMD4B*	5upstream	T	0.111	0.146

^a^ Physical location of SNPs based on human genome build 37 (GRCh37).

^b^ Minor allele frequency in normal Japanese of the 1000 Genomes database.

^c^ Minor allele frequency in 72 BCP‐ALL cell lines established from Japanese patients.

The genome‐wide association analysis between 34 Dex‐resistant cell lines (the IC50 value of Dex > 200 nmol/L) and 38 Dex‐sensitive cell lines (the IC50 value of Dex < 200 nmol/L) showed no marked associations (Figure S2).

### 3.3 Association between *FRMD4B*, *MITF* and *TGFBR3* and glucocorticoid sensitivity

**Figure 3:**
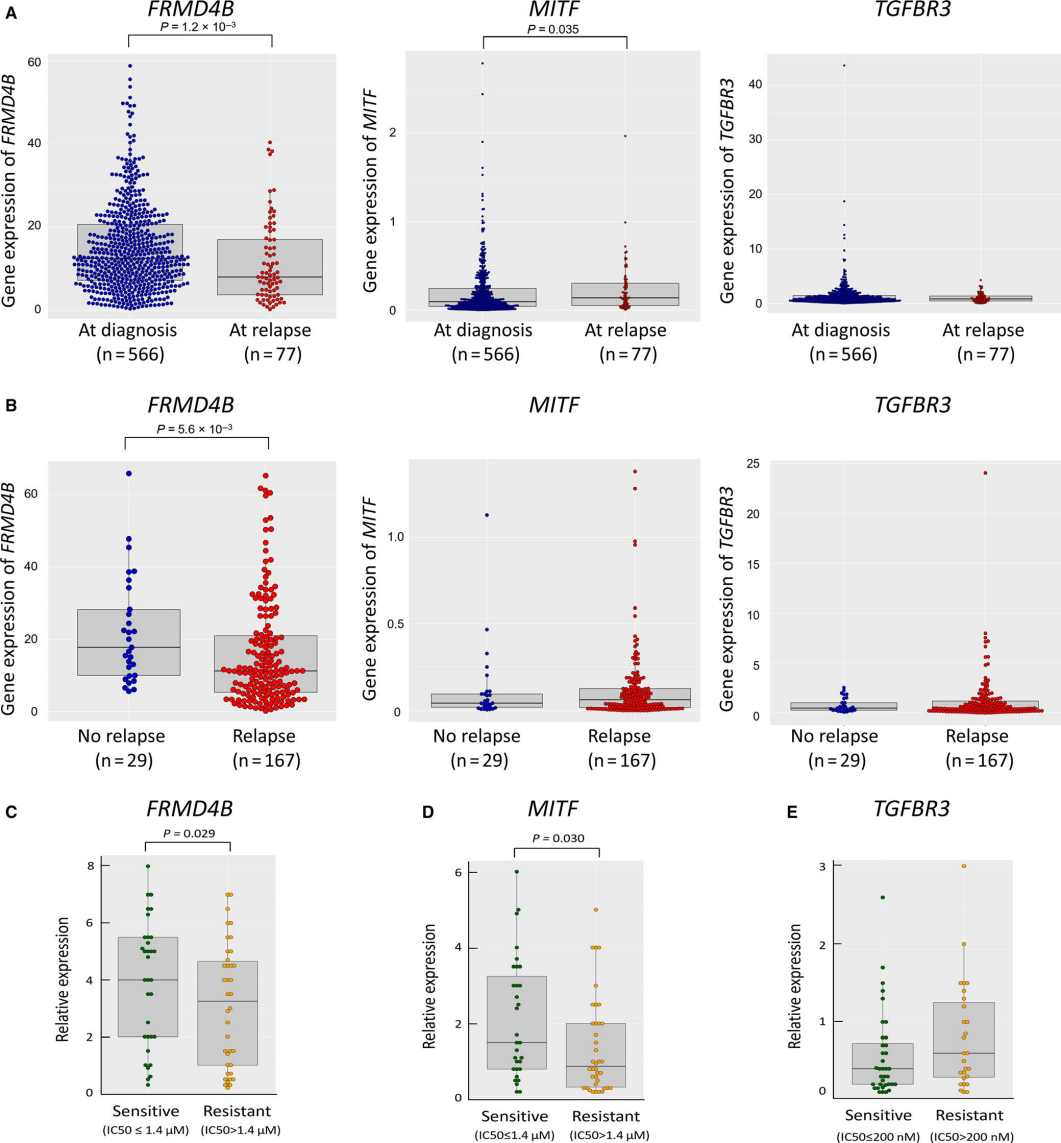
Association of *FRMD4B*, *MITF*, and *TGFBR3* with glucocorticoid sensitivity

To examine the potential clinical significance of the *FRMD4B*, *MITF* and *TGFBR3* genes in glucocorticoid sensitivity, we first analysed two childhood BCP‐ALL cohort databases. It is well known that glucocorticoid sensitivity both in vivo^40^ and in vitro^17^, ^41^ is highly associated with therapeutic outcome in childhood BCP‐ALL. In particular, it was reported that in vitro IC50 values of Pred in the ALL samples at relapse (median: 2370 μmol/L) were approximately 360 times higher than those in the ALL samples at initial diagnosis (median: 6.7 μmol/L).^42^ Notably, in the St. Jude PeCan database, gene expression levels of *FRMD4B*, but not *MITF* and *TGFBR3*, in BCP‐ALL samples at relapse (n = 77) were significantly lower than those at initial diagnosis (n = 566; Figure 3A). Similarly, in the TARGET database, gene expression level of *FRMD4B*, but not *MITF* and *TGFBR3*, in BCP‐ALL patients with relapse (n = 167) was significantly lower than those without relapse (n = 29; Figure 3B).

Next, we performed real‐time RT‐PCR analyses of the *FRMD4B*, *MITF* and *TGFBR3* genes in 72 BCP‐ALL cell lines. Gene expression levels of *FRMD4B*, *MITF* and *TGFBR3* were unchanged in BCP‐ALL cell lines after treatment with 100 nmol/L of Dex for 12 hours (Figure S3). However, baseline gene expression levels of *FRMD4B* and *MITF* in 38 Pred‐resistant cell lines (IC50 of Pred > 1.4 μmol/L) were significantly (*P* = .029 and .031, respectively) lower than those in 34 Pred‐sensitive cell lines (Figure 3C,D). In contrast, no significant differences were observed in baseline gene expression levels for *TGFBR3* between 34 Dex‐resistant cell lines (IC50 of Dex > 200 nmol/L) and 38 Dex‐sensitive cell lines (Figure 3E).

### 3.4 Association between genetic variants and glucocorticoid receptor gene expression

**Figure 4:**
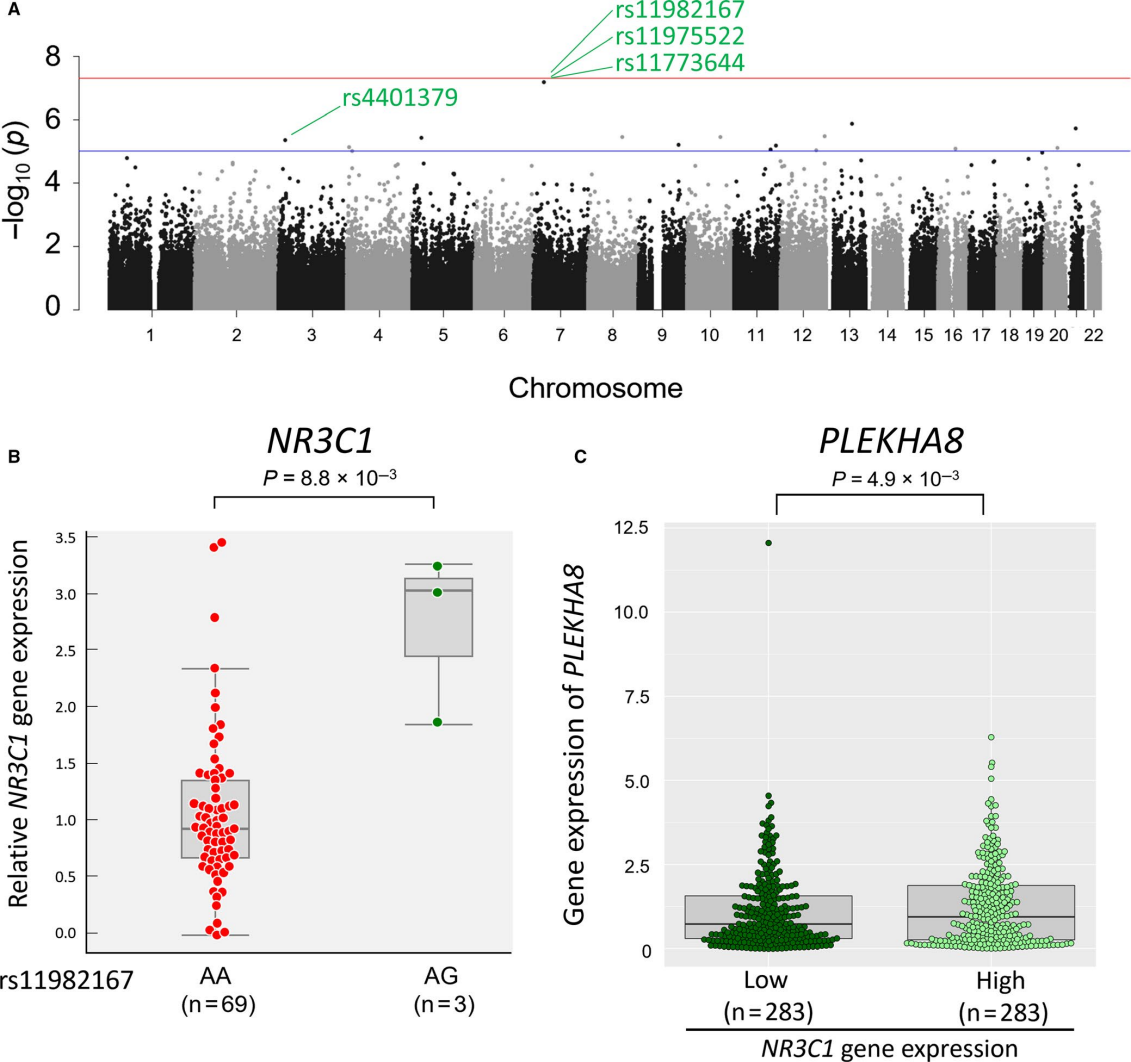
Association of inherited genetic variants with glucocorticoid receptor gene expression

In our examination of genome‐wide SNPs and *NR3C1* gene expression, we used measures quantified by the real‐time RT‐PCR analysis with primers for exon 8 and 9a that were specific for *GRα*, *GRγ* and *GR‐A*, as they had the highest negative correlation with the IC50 of Pred (*R* ^2^ = 0.34) and Dex (*R* ^2^ = 0.28) in our previous study.^19^ The overall genome‐wide analysis showed no evidence of genomic inflation (λ = 1.00). No SNPs reached the genome‐wide significance threshold, but 20 SNPs showed suggestive associations (*P* < 10^−5^; Figure 4A and Table 3). There were 3 borderline genome‐wide significant SNPs in complete LD (rs11982167, rs11975522 and rs11773644, *P* = 6.44 × 10^−8^) located at 7p14.3 and annotated to the *PLEKHA8* gene. Median relative gene expression levels of *NR3C1* by rs11982167 AA (n = 69) and GA (n = 3) genotypes were 0.93 and 3.02, respectively (Figure 4B). To further verify the association between the *PLEKHA8* gene and *NR3C1* gene expression in BCP‐ALL, we evaluated the gene expression profile in the St. Jude PeCan database. BCP‐ALL samples (n = 566) were divided into 283 samples showing higher than median *NR3C1* expression levels and 283 samples with lower than median *NR3C1* expression levels at diagnosis. We observed statistically significant higher levels of *PLEKHA8* gene expression among samples in the high category of *NR3C1* gene expression compared to the low category (*P* = .026; Figure 4C).

**Table 3:** Top SNPs associated with glucocorticoid receptor gene expression

SNP	Chr	Position^a^	*P* value	Gene	SNP function	Minor allele (JPT)	MAF	
							JPT^b^	Cell line^c^
rs11982167	7	30128903	6.44 × 10^−8^	*PLEKHA8*	Intronic	G	0.064	0.021
rs11975522	7	30129125	6.44 × 10^−8^	*PLEKHA8*	Intronic	A	0.064	0.021
rs11773644	7	30131289	6.44 × 10^−8^	*PLEKHA8*	3downstream	G	0.064	0.021
rs12875327	13	75314681	1.35 × 10^−6^			C	0.25	0.201
rs11842690	13	75315873	1.35 × 10^−6^			G	0.207	0.201
rs1327730	13	75318079	1.35 × 10^−6^			A	0.25	0.201
rs219649	21	27824285	1.86 × 10^−6^	*CYYR1‐AS1*	Intronic	A	0.3	0.319
rs7136734	12	129223121	3.3 × 10^−6^	*LOC102723323*		G,T	0.180	0.139
rs3824145	8	99140662	3.44 × 10^−6^	*POP1*	Coding	A,T	0.03	0.014
rs9332146	10	96722244	3.44 × 10^−6^	*CYP2C9*	Intronic	A	0.012	0.014
rs4565182	5	26372563	3.65 × 10^−6^			G,T	0.033	0.056
rs4401379	3	18930125	4.41 × 10^−6^	*AC144521.1*	Intronic	T	0.128	0.104
rs10982218	9	117200140	6.22 × 10^−6^	*DFNB31*	Intronic	A,C	0.017	0.028
rs2852835	11	121879938	6.45 × 10^−6^			C	0.267	0.174
rs7675928	4	6890937	7.31 × 10^−6^			T	0.064	0.049
rs10485674	20	39293832	7.92 × 10^−6^			G	0.017	0.014
rs7187736	16	51488206	8.22 × 10^−6^			G	0.087	0.049
rs12421832	11	106465539	8.73 × 10^−6^			G	0.01	0.021
rs7975754	12	105328199	9.35 × 10^−6^	*SLC41A2*	Intronic	C	0.25	0.236
rs3763969	4	16648246	9.56 × 10^−6^	*LDB2*	Intronic	G,T	0.122	0.09

^a^ Physical location of SNPs based on human genome build 37 (GRCh37).

^b^ Minor allele frequency in normal Japanese of the 1000 Genomes database.

^c^ Minor allele frequency in 72 BCP‐ALL cell lines established from Japanese patients.

### 3.5 Correlation between genetic variants associated with prednisolone sensitivity and those with glucocorticoid receptor gene expression

**Figure 5:**
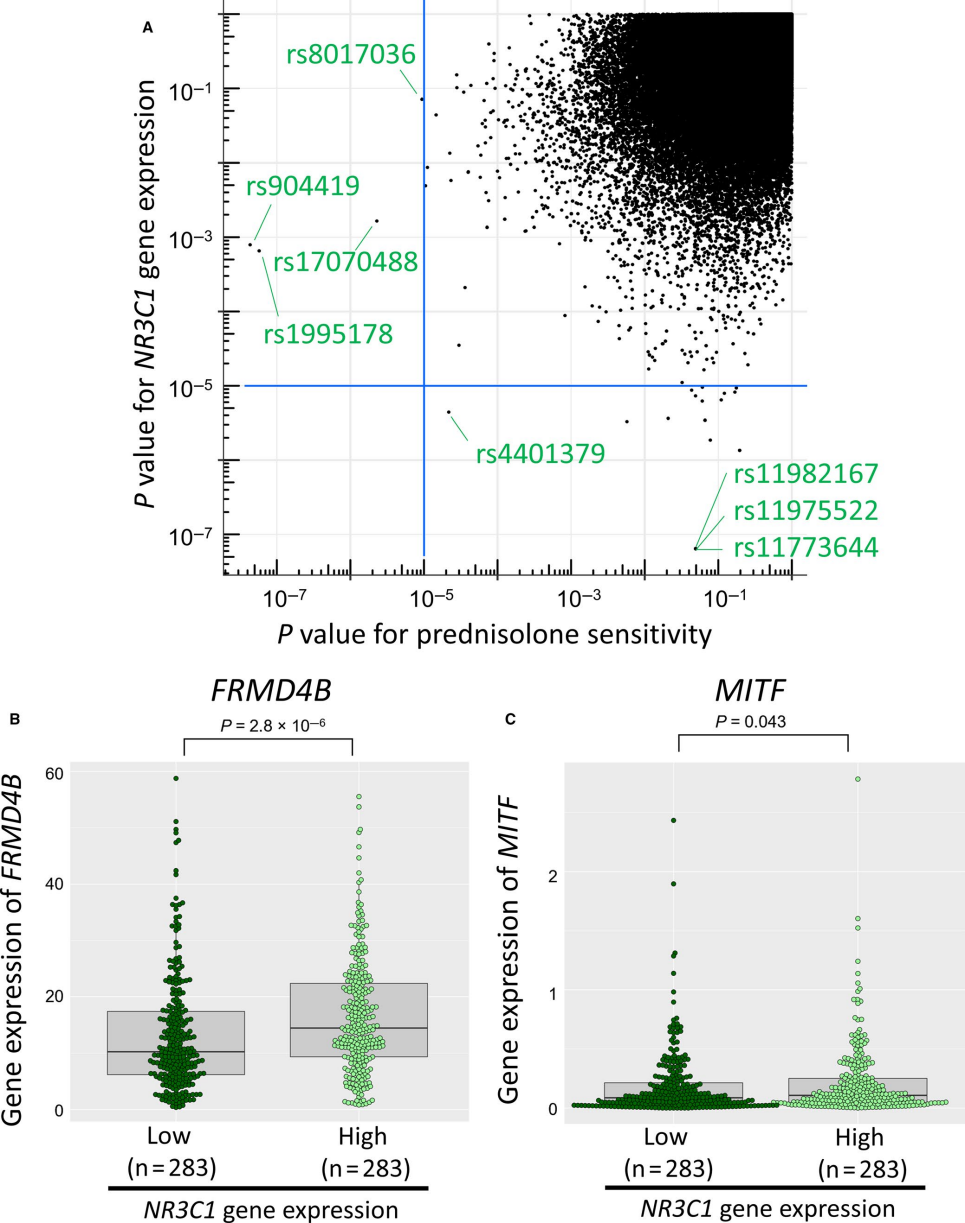
Correlation between inherited genetic variants associated with prednisolone sensitivity and those with glucocorticoid receptor gene expression

As GR expression is critically involved in the GC sensitivity of ALL, we examined patterns of potential concordance in association between the genome‐wide results of *NR3C1* gene expression and Pred sensitivity (Figure 5A). The two genome‐wide significant SNPs (rs904419 and rs1995178 located between the *FRMD4B* and *MITF* genes) observed in the Pred sensitivity analysis showed indication of an association with *NR3C1* gene expression association as well (*P* = 7.90 × 10^−4^ and 6.54 × 10^−4^, respectively). Consistent with this finding, among BCP‐ALL samples at diagnosis in the St. Jude PeCan database, gene expression levels of *FRMD4B* and *MITF* were significantly higher in samples that showed higher levels of *NR3C1* gene expression (Figure 5B). In contrast, three borderline significant SNPs in the *PLEKHA8* gene (rs11982167, rs11975522 and rs11773644) associated with *NR3C1* gene expression (*P* = 6.44 × 10^−8^) showed weak evidence of association with Pred sensitivity (*P* = 4.92 × 10^−2^). In the TARGET and St. Jude PeCan databases, gene expression levels of *PLEKHA8* were not associated with disease relapse in childhood BCP‐ALL (Figure S4). In addition, rs4401379 (located at 3p24.3) showed concordance in potential association with both Pred sensitivity (*P* = 2.17 × 10^−5^) and *NR3C1* gene expression (*P* = 4.41 × 10^−6^).

## 4 DISCUSSION

Identification of genetic variants that affect the tumour cells’ sensitivity to chemotherapeutic agents may indicate novel drug targets. Differences in pharmacokinetics are critically involved in the inter‐individual difference in drug response.^1^, ^2^, ^3^ Using in vitro drug sensitivity measures in tumour cell lines can be considered a more direct approach to identifying associated genetic variants compared to examining clinical responses in patients which are often confounded by individual circumstance. In the present study, we examined genome‐wide SNP associations with in vitro GC sensitivities in 72 leukaemic cell lines established from an ethnically homogeneous population of Japanese BCP‐ALL patients.

The top genome‐wide significant SNP associated with Pred sensitivity based on log IC50 values was rs904419 located at 3p14.1. This SNP also showed the strongest association in the GWAS that examined Pred sensitivity as a binary outcome. rs904419 is located between the *FRMD4B* and *MITF* genes, suggesting a role for either or both of these genes in Pred sensitivity of BCP‐ALL. Moreover, we observed two lines of corroborating evidence; (a) from two childhood BCP‐ALL databases showing lower gene expression levels of *FRMD4B* associated with disease relapse and (b) lower gene expression levels of these genes associated with Pred resistance in real‐time RT‐PCR analysis of our BCP‐ALL cell lines. The protein encoded by the *FRMD4B* (*GRSP1*) gene is a GRP1‐binding protein, which may act as a scaffolding protein, as it contains both two coiled coil domains and a FERM protein interaction domain.^43^ Association of *FRMD4B* genetic variants had been reported in GWAS of advanced heart failure,^44^ coeliac disease^45^ and type 1 autoimmune pancreatitis.^46^ Interestingly, GWAS of thiopurine metabolism in UK ALL97 patients identified an association with *FRMD4B* genetic variants.^47^ Notably, knockdown of *FRMD4B* by siRNA has been reported to induce significant resistance to thiopurine in U251 (human glioma) and Hela (human cervical carcinoma) cell lines.^46^ Regarding the *MITF* gene, it may encode a transcription factor, as it contains both a leucine zipper domain and basic helix‐loop‐helix domain.^48^ MITF is involved in melanocyte development by transcriptionally regulating melanogenesis‐specific gene expression including genes involved in cell proliferation and survival.^48^ It should be noted that knockdown of MITF by shRNA has been reported to promote apoptosis in multiple myeloma cells treated with dexamethasone.^49^ These observations suggested that the identified genetic variants may be associated with Pred sensitivity in BCP‐ALL cells through regulation of apoptosis and/or cell proliferation by FRMD4B, MITF or both.

The Dex sensitivity analysis using log IC50 values showed suggestive associations with rs2306888 located at 1p22.1 and annotated to the *TGFBR3* gene which encodes the type III receptor of TGF‐beta. However, the association was attenuated in the GWAS of Dex sensitivity as a binary outcome. rs2306888 is a synonymous SNP at codon 173 of the *TGFBR3* gene (TCA > TCG). Synonymous (silent) SNPs may affect protein expression through stability and subcellular localization of messenger RNA.^2^ However, in two childhood BCP‐ALL databases, gene expression levels of *TGFBR3* were not associated with disease relapse, and, in BCP‐ALL cell lines, basal gene expression levels were not associated with Dex sensitivity. TGF‐beta type III receptor forms complex with other members of TGF‐beta receptor superfamily.^50^ Furthermore, soluble TGFBR3 produced by ectodomain shedding may suppress signalling of TGFB pathway.^50^ TGFBR3 expression level has been reported to be decreased in a wide variety of cancers.^50^ *TGFBR3* genetic variant associations have been reported in GWAS of pulmonary emphysema,^51^ bone mineral density,^52^, ^53^ damage to the optic nerve due to glaucoma^54^, ^55^ and testicular dysgenesis syndrome.^56^, ^57^ Although functional association of TGFBR3 with GC sensitivity is unclear, GC treatment has been shown to up‐regulate TGFBR3 expression in osteoblast‐like cells^58^ and hepatic stellate cells.^59^ These previous reports suggest a possible involvement of the *TGFBR3* gene in GC sensitivity as a downstream target at least in certain types of cells.

In addition to germline genetic polymorphisms, somatic mutations and epigenetic modification can alter the drug sensitivity of leukaemia cells.^2^ Maranville et al^60^ recently reported GWAS results of Dex sensitivity in phytohemagglutinin‐stimulated (PHA‐stimulated) peripheral‐blood lymphocytes of 88 African‐American healthy donors. rs11129354 showed genome‐wide significance (*P* = 4 × 10^−8^) with per cent inhibition at 1 µmol/L of Dex.^60^ rs11129354 is located at 3p24.1 and 5′ upstream region of the *RBMS3* gene. Of note, although genetic background and GC sensitivity measurement approach was not identical, rs11129354 was not associated with Pred (*P* = .242) and Dex (*P* = .211) sensitivity in our study. This difference between PHA‐stimulated normal lymphocytes and ALL cell lines suggests that acquired somatic mutations and epigenetic changes may alter the significance of germline genetic variants on GC sensitivity of leukaemia cells from that of normal lymphocytes. Also, it is possible that the variant identified in African Americans may not be tagging the functional locus in the same way in Japanese.

In our analysis of genome‐wide SNPs and *NR3C1* gene expression levels, we observed the strongest associations for rs11982167, rs11975522 and rs11773644, located at 7p14.3 and annotated to *PLEKHA8* gene. Evaluation using the St. Jude PeCan database of childhood BCP‐ALL samples showed supportive evidence in which higher gene expression levels of *PLEKHA8* was associated with higher *NR3C1* gene expression. *PLEKHA8* (FAPP2) gene encodes a cytosolic protein that has a potential to transfer glucosylceramide.^61^ Association with genetic variants of the *PLEKHA8* gene had not been reported in previous GWAS. Thus, functional consequences of *PLEKHA8* and *NR3C1* in BCP‐ALL remain unclear.

In interpreting the difference in results between the analysis of Pred and Dex, it is important to acknowledge that the ranges of concentration for drug sensitivity measurements were different between the two; the upper limit concentration of Pred (417 µmol/L) was more than 1000 times higher than that of Dex (250 nmol/L). These measurement distributions may have had variable influence on the stability of the regression modelling used in the genome‐wide association analysis, which may partly explain the observed differences in top ranked loci between the Pred sensitivity (*FRMD4B* and *MITF*) and Dex sensitivity (*TGFBR3*) analyses. To address this limitation and increase the confidence in our findings, we identified corroborating evidence through in silico approaches that accessed external databases, as well as laboratory follow‐up for these target loci. In addition, we supplemented the genome‐wide analysis approach using logistic regression after dichotomizing samples based on drug sensitivity value thresholds. Although results appeared to be attenuated, the top associated loci in the Pred analysis was consistent with the analysis in which drug sensitivities were treated as a quantitative trait.

In conclusion, we demonstrated that a GWAS of in vitro drug sensitivity in a panel of leukaemia cell lines may be useful in identifying germline variants associated with drug sensitivity of leukaemia cells. Clinical significance of the germline variants identified in the present study warrants validation in a separate study of patient samples. Germline variants that influence drug sensitivity of leukaemia cells may give rise to personalized medicine for effective and safe chemotherapy in leukaemia patients.

## CONFLICTS OF INTEREST

The authors confirm that there are no conflicts of interest.

## AUTHOR CONTRIBUTIONS

**Tamao Shinohara:** Conceptualization (lead); data curation (lead); formal analysis (lead); investigation (lead); methodology (lead); project administration (lead); resources (lead); software (equal); supervision (lead); validation (lead); visualization (lead); writing‐original draft (lead); writing‐review & editing (lead). **Kevin Urayama:** Data curation (lead); formal analysis (lead); validation (lead); visualization (lead); writing‐review & editing (lead). **Atsushi Watanabe:** Conceptualization (lead); data curation (lead); formal analysis (lead); investigation (lead); methodology (lead); project administration (lead); software (lead); supervision (lead); validation (lead); visualization (lead); writing‐original draft (equal); writing‐review & editing (lead). **Koshi Akahane:** Conceptualization (supporting); writing‐review & editing (supporting). **Kumiko Goi:** Conceptualization (supporting); writing‐review & editing (supporting). **MEIXIAN HUANG:** Conceptualization (supporting); writing‐review & editing (supporting). **Keiko Kagami:** Data curation (supporting); formal analysis (supporting); Writing‐review & editing (supporting). **Masako Abe:** Data curation (supporting); formal analysis (supporting); writing‐review & editing (supporting). **Kanji Sugita:** Conceptualization (supporting); writing‐review & editing (supporting). **Yukinori Okada:** Conceptualization (supporting); data curation (supporting); formal analysis (supporting); supervision (supporting); writing‐review & editing (supporting). **Hiroaki Goto:** Data curation (supporting); writing‐review & editing (supporting). **Masayoshi Minegishi:** Data curation (supporting); writing‐review & editing (supporting). **Shotaro Iwamoto:** Data curation (supporting); writing‐review & editing (supporting). **Takeshi Inukai:** Conceptualization (lead); data curation (lead); formal analysis (lead); funding acquisition (lead); investigation (lead); methodology (lead); project administration (lead); resources (lead); software (lead); supervision (lead); validation (lead); visualization (lead); writing‐original draft (equal); writing‐review & editing (lead).

## Supporting information

Fig S1‐S4

## Data Availability

All data relevant to the study are included in the article.
