# Supplementary figures and images for: Inherited genetic variants associated with glucocorticoid sensitivity in leukaemia cells
Source: J Cell Mol Med. 2020 Oct 1;24(22):12920–32. doi: 10.1111/jcmm.15882 (PMC7701530; doi:10.1111/jcmm.15882)

**Supplemental Figure 1**

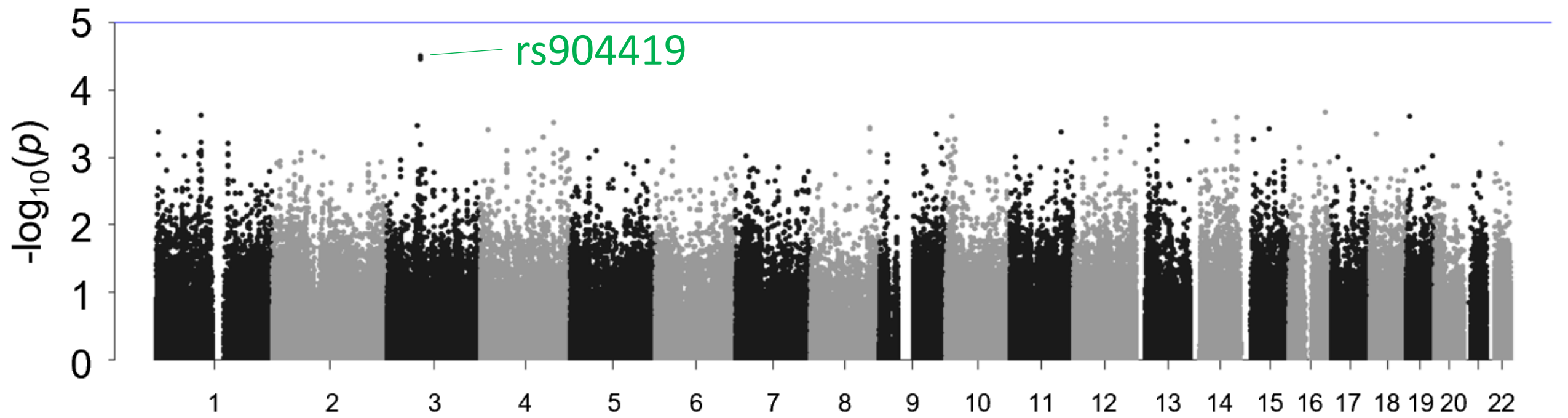

**Supplemental Figure 2**

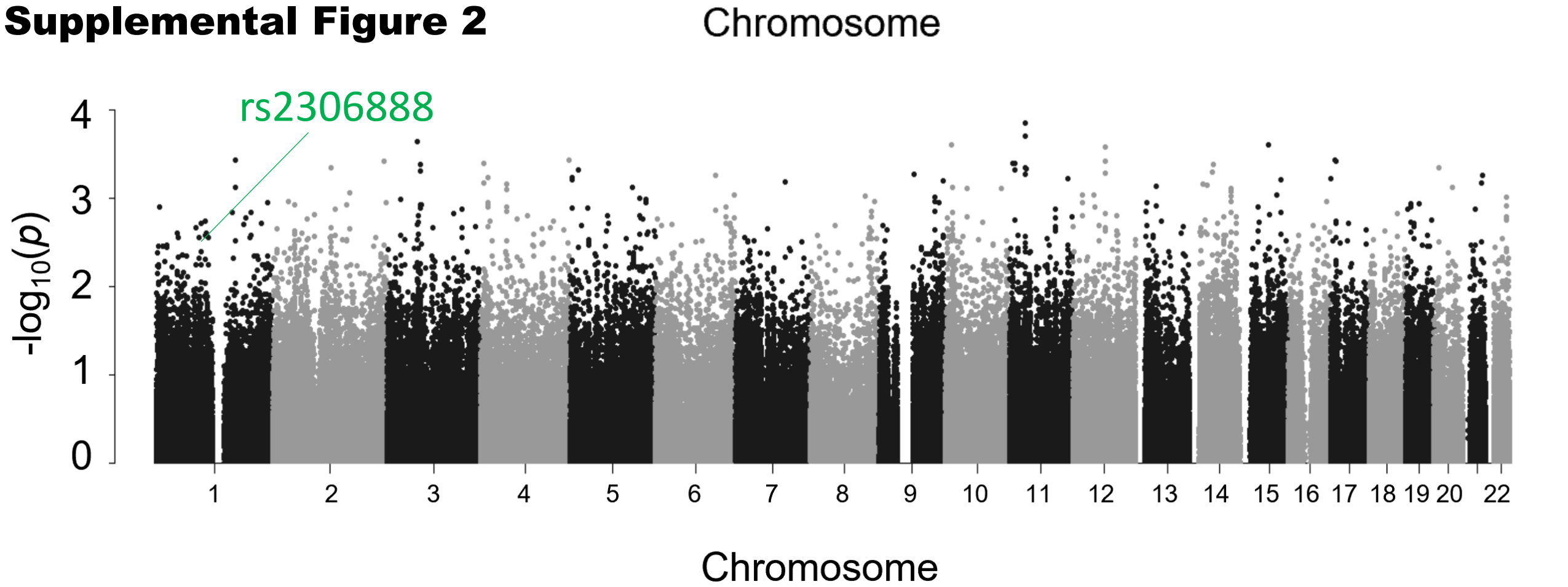

**Supplemental Figure 3**

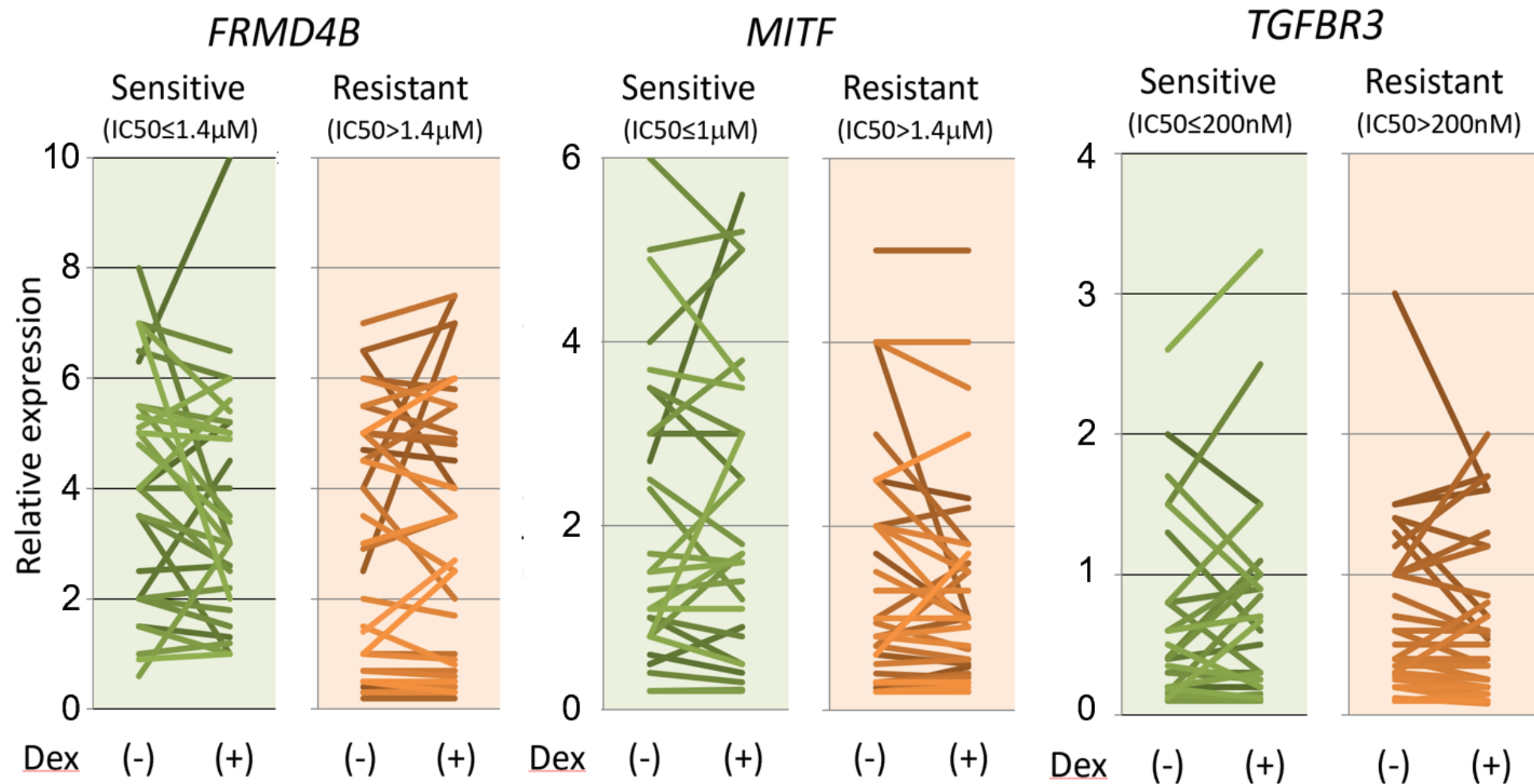

Supplemental Figure 4

*PLEKHA8*

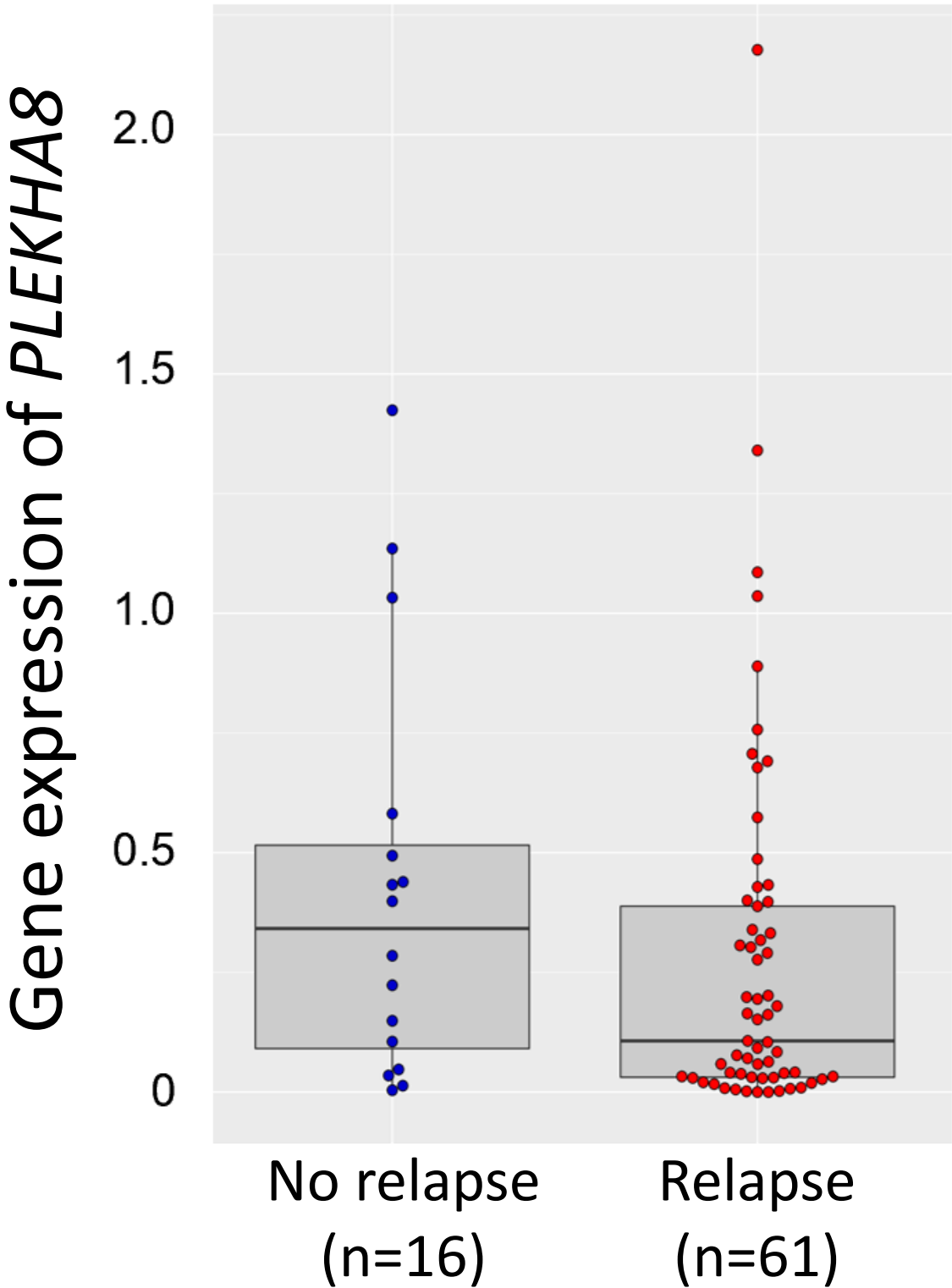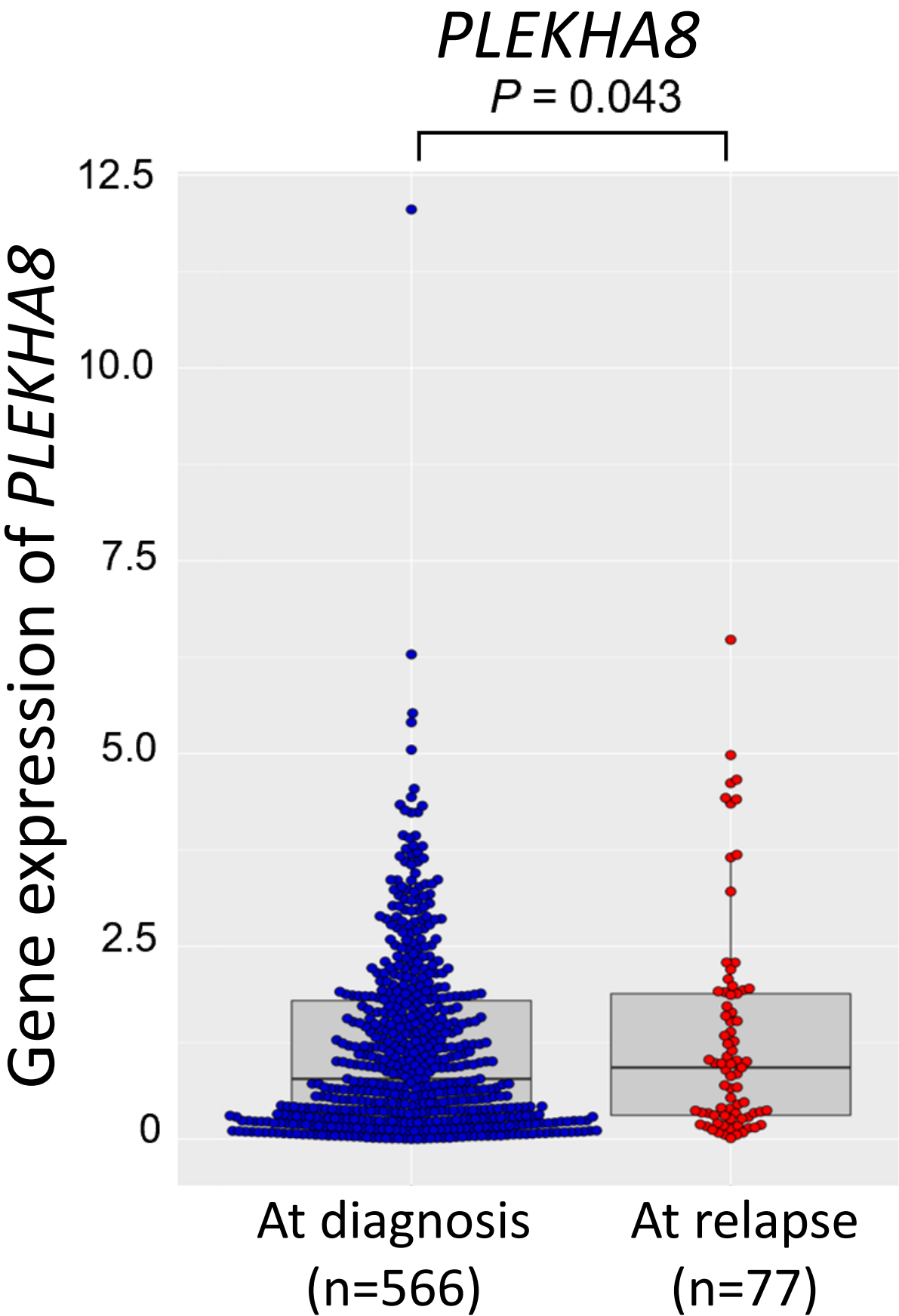

Supplement: Supplementary file 1 — Fig S1‐S4 [file JCMM-24-12920-s001.pdf]
